# Enzyme-Linked Immunosorbent Assay for the Detection of Severe Acute Respiratory Syndrome Coronavirus 2 (SARS-CoV-2) IgM/IgA and IgG Antibodies Among Healthcare Workers

**DOI:** 10.7759/cureus.10285

**Published:** 2020-09-06

**Authors:** Suliman A Alharbi, Abdullah Z Almutairi, Abdulhalem A Jan, Amal M Alkhalify

**Affiliations:** 1 Laboratory and Blood Bank - Immunology, Serology and Tissue Typing Department, King Fahad General Hospital, Madinah, SAU; 2 Laboratory and Blood Bank - Microbiology Department, King Fahad General Hospital, Madinah, SAU; 3 Laboratory and Blood Bank - Hematology Department, King Fahad General Hospital, Madinah, SAU

**Keywords:** elisa, sars-cov-2, covid-19, hcws, seroprevalence, anti-sars-cov-2

## Abstract

Background

The outbreak of the novel coronavirus disease 2019 (COVID-19), caused by the severe acute respiratory syndrome coronavirus 2 (SARS-CoV-2), has been spreading rapidly across the world. A nucleic acid real-time quantitative polymerase chain reaction (RQ-PCR) test of nasopharyngeal samples is the standard method for the diagnosis of an active SARS-CoV-2 infection. However, many limitations of the RQ-PCR tests make them unsuitable for the simple and rapid diagnosis of COVID-19 patients. Moreover, some individuals with COVID-19 present an asymptomatic infection. Thus, assessing the asymptomatic transmission of COVID-19, especially in healthcare workers (HCWs), is crucial for evaluating the efficiency of the current preventive measures. Serological tests such as enzyme-linked immunosorbent assay (ELISA) are needed to quickly identify a large number of asymptomatic carriers to prevent the further spread of the virus and assess level of possible serological immunity in a community.

Method

Between April 18 and June 17, 2020, 330 HCWs from five Madinah region-affiliated hospitals underwent a seroprevalence screening for anti-SARS-CoV-2 antibodies (immunoglobulin [Ig]M/IgA and IgG) using indirect ELISA testing.

Result

Among the 330 samples, 80 (24.24%) were positive for SARS-CoV-2 IgM/IgA and/or IgG antibodies. There were no significant differences observed in the seroprevalence among the different occupations of the HCWs (excluding the pharmacists) with respect to the percentage of their seropositive samples.

Conclusion

The current study presented the seroprevalence of anti-SARS-CoV-2 IgM/IgA and IgG antibodies in HCWs. The regular screening of HCWs for these antibodies is necessary; subsequently, a molecular test is recommended for those with seropositive (IgM, IgA, and IgG) samples to assess their viral load and potential shedding.

## Introduction

A series of pneumonia cases of an unknown cause emerged at the end of 2019 in Wuhan, a city in China's Hubei Province [1]. In January 2020, the causative agent for the pneumonia cases was identified as the severe acute respiratory syndrome coronavirus 2 (SARS-CoV-2). The novel virus was identified using a deep sequencing analysis of the lower respiratory tract samples [2].

The World Health Organization named the disease coronavirus disease 2019 or “COVID-19” and on March 11, 2020 declared it a pandemic [3]. Currently, the virus is spread across 215 countries; the number of infections is more than 30,399,907 and the number of deaths is over 971,452 [4].

In SARS-CoV-2, it is difficult to differentiate between healthy individuals and COVID-19 cases [5]. The commonly reported clinical symptoms of confirmed COVID-19 cases include cough, fever, fatigue, and myalgia [2]. However, these symptoms are similar to that of other viral diseases such as influenza [6] and are therefore not unique features of COVID-19.

Moreover, COVID-19 may be associated with an asymptomatic or pre-symptomatic disease transmission. Parameter values measure the relative contribution of the transmission of COVID-19 from asymptomatic individuals (do not exhibit symptoms during the course of infection) compared to transmission of COVID-19 from pre-symptomatic individuals (do not exhibit symptoms at the time of testing but later during the course of infection). However, the percentage of asymptomatic individuals is difficult to estimate as these individuals do not know that they are infected without being examined, typically through a scientific study [7]. 

Currently, the primary tools for clinical diagnosis of COVID-19 infection include identifying some hematological parameters, performing computed tomography (CT) imaging, and virus nucleic acid real-time polymerase chain reaction (RT-PCR) testing [8]. However, there are many limitations of the RT-PCR tests that make them unsuitable for use in the field for simple and rapid screening of patients such as complex protocols, number of false negatives, and long turned turnaround times (TAT) [9].

Meanwhile, indirect detection methods such as serological tests can detect a waning or previous COVID-19 infection by measuring the host humoral immune response to the virus. While direct detection methods usually remain the primary tool for diagnosing an active COVID-19 infection, the use of indirect methods such as serological assays has several important applications in responding to and monitoring the COVID-19 pandemic [10]. Presently, these serological tests cannot be used to determine whether an individual is immune or not; however, these tests can provide information about populations that may be immune and thus potentially protected. These tests can also help in determining the proportion of a population previously infected with COVID-19, the communities that have experienced the highest rate of infection (may show higher rates of herd immunity), potentially infected individuals, and individuals who may qualify as blood donors of convalescent plasma to be used in the emergency treatment of COVID-19 [10].

Healthcare workers are the frontline workforce in hospitals that deal with COVID-19 patients. Thus, they are a high-risk population and therefore studies related to their seroprevalence, asymptomatic cases, seroconversion, and disease transmission are crucial.

Hence, the aim of our study was to identify the cases of asymptomatic COVID-19 infections among healthcare workers using serological tests to detect the seroprevalence of immunoglobulin [Ig]M/IgA and IgG antibodies against SARS-CoV-2.

## Materials and methods

Collection of samples

This study has been approved by the Institutional Review Board of the General Directorate of Health Affairs, Madinah, Kingdom of Saudi Arabia (IRB: 442). A total of 330 blood samples were collected from healthcare workers (HCWs; those who did not have overt symptoms) including physicians, nurses, laboratory technologists, pharmacists, infection control staff, and administrative staff working in different wards in five Madinah region-affiliated hospitals between April 28, 2020 and June 17, 2020. The serum samples were de-identified, inactivated at 56°C for 30 min, and stored at -70°C until use.

Enzyme-linked immunosorbent assays

The samples were tested for SARS-CoV-2 antibodies IgM/IgA (# MA1032) and IgG (# G1032) using indirect enzyme-linked immunosorbent assays (ELISA) kits purchased from Vircell Microbiologists (Granada, Spain). Both kits use SARS-CoV-2 recombinant antigens: spike glycoprotein (S protein) and nucleocapsid (N protein). 

The tests were performed strictly according to the manufacturer’s instructions. For the detection of the IgM/IgA antibodies, 25 μL of IgG ELISA sorbent (# S001, Vircell Microbiologists), 5 μL of the serum sample, and 75 μL of the serum diluent were added to the wells (total volume: 105 μL/well). For the detection of the IgG antibody, 5 μL of serum sample was added to 95 μL of serum diluent to produce a 1/20 dilution. Then, 20 μL of the diluted samples were added to 80 μL of the serum diluent (total volume: 100 μL/well). Next, the plates were incubated at 37°C for 45 min followed by washing. After washing, 100 μL of the antibody-conjugate was added to the respective wells and the plates were incubated at 37°C for 30 min. After the second washing 100 μL of the substrate was added and the plates were incubated at room temperature for 20 min in the dark. Finally, the reaction was stopped by adding 50 μL of stop solution. In both the ELISA tests, 100 μL of the positive control, negative control, and cut-off control (in duplicates) were used. The optical density (OD) readings were taken at 450 nm (620 nm as a reference filter) in the BEP III System (ELISA analyzer; Siemens HealthCare Diagnostics, Marburg, Germany). 

Interpretation of the ELISA results

The antibody index for the ELISA tests was calculated as per the formula outlined in the technical brochure of the kit: antibody index = (sample OD)/(cut-off serum mean OD) × 10.

Further, the test quality was determined by the following criteria: the measured absorbance value must be ≥0.9 for the positive control, <0.5 for the negative control, and between 0.55 and 1.5 for the cut-off control. The test was invalidated if any one of these criteria were not met.

For anti-SARS-CoV-2 IgM/IgA, an antibody index of <6 was considered negative, between 6 and 8 was considered equivocal (needed repeat testing), and >8 was considered positive for IgM/IgA specific antibodies against SARS-CoV-2. Whereas, for anti-SARS-CoV-2 IgG, an antibody index of <4 was considered negative, between 4 and 6 was considered equivocal, and >6 was considered positive for IgG specific antibodies against SARS-CoV-2.

Performance of the ELISA test kit

The manufacturer's performance characteristics were available prior to the study; the manufacturers had assayed a total of 1479 samples, including 286 pre-pandemic samples were selected from healthy donors and 1193 samples collected from hospitalized patients (post-PCR). 

Moreover, the sensitivity and specificity of the IgG kit have been assessed by Kohmer et al. [11], where they tested 33 positive samples for sensitivity and 21 human coronavirus 229E (HCoV-229E) samples for specificity.

Further, we conducted additional sensitivity and specificity testing of these kits by using the sera from 40 RT-PCR positive samples and 65 (10 of which were HIV-positive) pre-COVID-19 samples collected from King Fahad Hospital, Madinah.

Statistical analysis

The Statistical Package for the Social Sciences (SPSS) v24 (IBM Corp., Armonk, NY, USA) software was used to perform the statistical calculations. A chi-square goodness of fit test was used to compare the percentage of all the seropositive samples for the same occupation and the sum of the percentages of all seropositive samples for other occupations.

## Results

Sensitivity and specificity of the VIRCELL ELISA test kits

To test the sensitivity and specificity of IgM/IgA and IgG SARS-CoV-2 antibody kits, we tested a total of 105 blood samples, including 40 samples from clinically confirmed SARS-CoV-2-infected patients and 65 samples from pre-pandemic patients. Of the 40 positive samples, 35 were seropositive for IgM/IgA and 37 were seropositive for IgG; this resulted in a sensitivity of 87.5% and 92.5%, respectively. Of the 65 negative samples, 63 tested negative for IgM/IgA and 61 tested negative for IgG; this resulted in a specificity of 97.0% and 94.0%, respectively (Table 1 C). 

**Table 1:** Sensitivity and specificity of the IgM/IgA and IgG enzyme-linked immunosorbent assays (ELISA) kits for coronavirus disease 2019 (COVID-19). A, IgM/IgA and IgG test kits performance evaluation by the manufacturers; B, IgG test kit performance evaluation by Kohmer et al. [11]; C, IgM/IgA and IgG test kits performance evaluation by our laboratory; *d, days, Ig: immunoglobulin

Test	Sensitivity (%)				Specificity (%)		
	A	B		C	A	B	C
		5-9 d	10-18 d				
IgM/IgA	66.0 % (787/1193)	…	…	87.5 % (35/40)	99.0 % (283/286)	…	97.0 % (63/65)
IgG	58.0 % (692/1193)	70.6 % (12/17)	100 % (16/16)	92.5 % (37/40)	98.0 % (280/286)	95.2 % (20/21)	94.0 % (61/65)

Further, we considered the assessment of the test kit undertaken by the manufacturers. A total of 1479 samples, 1193 from clinically confirmed SARS-CoV-2-infected patients and 286 from pre-pandemic patients, were tested by them. Of the 1193 positive samples, 787 were seropositive for IgM/IgA and 692 for IgG; this resulted in a sensitivity of 66.0% and 58.0%, respectively. Of the 286 negative samples, 283 tested negative for IgM/IgA and 280 tested negative for IgG; this resulted in a specificity of 99.0% and 98.0%, respectively (Table 1 A). 

Finally, we also considered previous studies by Kohmer et al. [11]. According to them, this test kit resulted in a sensitivity of 70.6% for the early phase (5-9 days) PCR-confirmed infection and 100% for the late phase (10-18 days) PCR-confirmed infection; moreover, 20 of the negative samples (HCoV-229E-positive) tested negative for IgG, resulting in a specificity 95.2% (Table 1 B).

A combination of the above data provided us with a combined sensitivity of 66.70% for IgM/IgA and 60.0% for IgG, and a specificity of 98.58% for IgM/IgA and 97.04% for IgG for the Vircell ELISA kits.

Seroprevalence of antibodies against SARS-CoV-2 in healthcare workers

**Figure 1:**
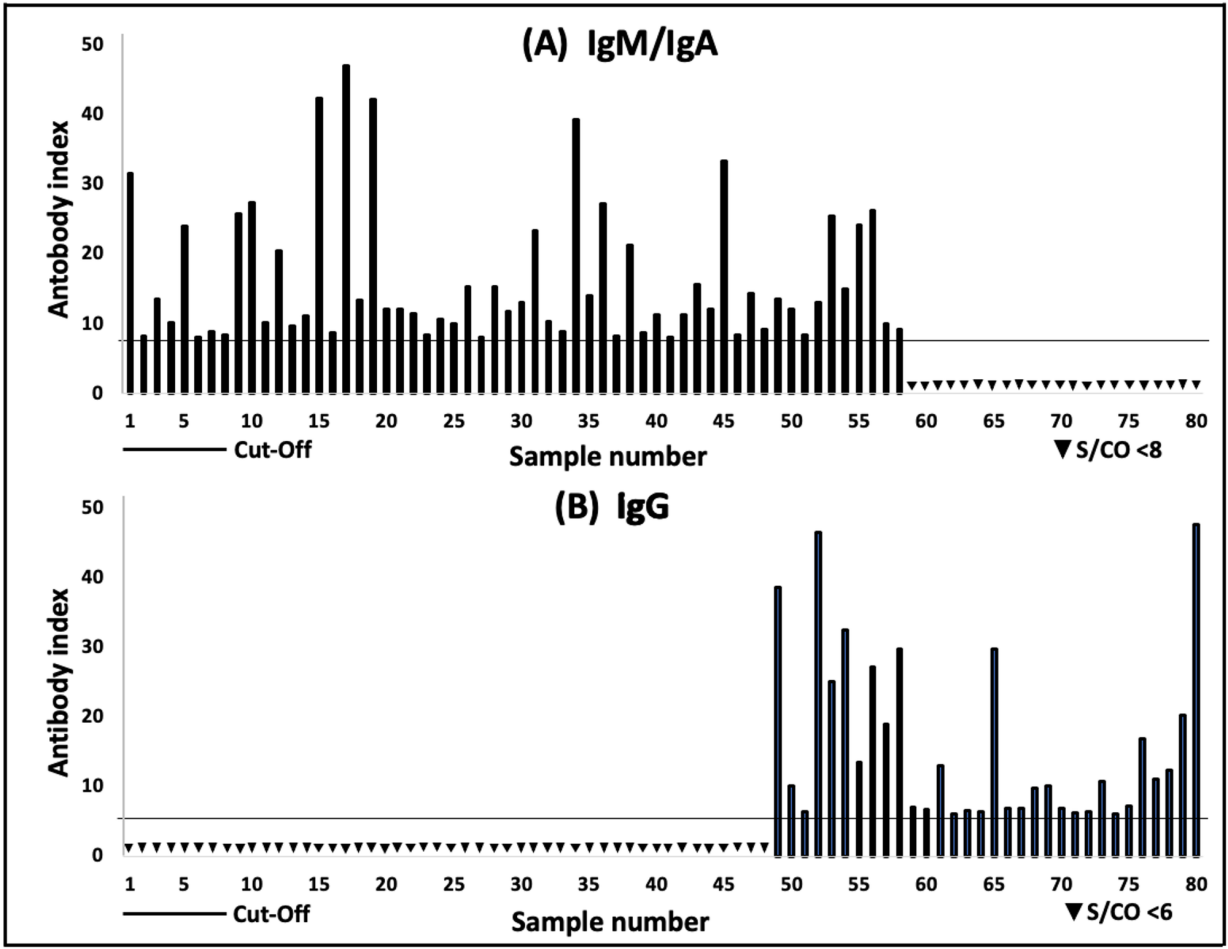
(A) Serological levels of positive severe acute respiratory syndrome coronavirus 2 (SARS-CoV-2)-specific IgM/IgA antibodies in samples from health care workers (HCWs), (B) Serological levels of positive SARS-CoV-2-specific IgG antibodies in samples from HCWs. The lanes mark of the seropositivity threshold. S/CO, signal-to-cutoff.

A total of 330 serum samples were collected from HCWs from five hospitals in Madinah and ELISA tests were performed on them for the detection of IgM/IgA and IgG antibodies against SARS-CoV-2. Out of these samples, 75.76% were negative for both IgM/IgA and IgG antibodies, 14.55% were positive for IgM/IgA and negative for IgG (Figure 1A, samples 1-48), 6.36% were positive for IgG and negative for IgM/IgA (Figure 1B, samples 59-80), while 3.33% were positive for both IgM/IgA and IgG as shown in samples 49-58 (Figure 1A, 1B). Therefore, the prevalence of infection in HCWs was 24.24% (80/330).

For the purpose of the analysis, we categorized the HCWs into groups as per their occupations. The distribution of the seropositive samples of the HCWs is shown in Table 2. In this study, the samples from the nurses represent 63.64% (210/330) of the total samples including 23.03% of nurses from emergency rooms (ERs), 21.21% from intensive care units (ICUs), 13.03% from airborne infection isolation rooms (AIIRs) and 6.36% from other hospital wards. The samples tested positive for IgM/IgA and negative for IgG in 17.11%, 18.57%, 18.60%, and 4.76% of the nurses from ERs, ICUs, AIIRs, and other wards, respectively. Further, the samples tested positive for IgG and negative for IgM/IgA in 6.58%, 4.29%, 6.98%, and 4.76% of the nurses from ERs, ICUs, AIIRs, and other wards, respectively. Finally, the samples tested positive for both IgM/IgA and IgG in 2.63%, 4.29%, 2.33%, and 4.76% of the nurses from ERs, ICUs, AIIRs, and other wards respectively (Table 2).

**Table 2:** Health care worker's occupation with the highest risk of COVID-19 infection. ER, emergency room; ICU, intensive care unit; AIIR, airborne infection isolation room; MLT, medical laboratory technologist; IC, infection control; Admin, administration; IgA, immunoglobulin A; IgM, immunoglobulin M; IgG, immunoglobulin G; N*, total number of samples; n, total number of positive samples.

Occupation	N^*^	IgM/IgA n (%)	IgG n (%)	IgM/IgA and IgG n (%)	Total % of positive samples	Chi-square Test value	P-value
ER nurses	76	13 (17.11)	5 (6.58)	2 (2.63)	26.32	9.42	0.224
ICU nurses	70	13 (18.57)	3 (4.29)	3 (4.29)	22.86		
AIIR nurses	43	8 (18.60)	3 (6.98)	1 (2.33)	27.91		
Other nurses	21	1 (4.76)	1 (4.76)	1 (4.76)	14.29		
Physicians	18	1 (5.56)	3 (16.67)	1 (5.56)	27.78		
MLTs	80	11 (13.75)	2 (2.50)	3 (3.75)	20.00		
Pharmacists	5	0 (0.0)	0 (0.0)	0 (0.0)	00.00		
IC staff	9	0 (0.0)	3 (33.33)	0 (0.0)	33.33		
Admin staff	8	1 (12.50)	1 (12.50)	0 (0.0)	25.00		

On the other hand, the rest of the HCW occupations represent 36.36% (120/330) of the total samples. These include 5.45% of physicians, 24.24% of medical laboratory technologists (MLTs), 1.52% of pharmacists, 2.73% of infection control (IC) staff, and 2.42% of administration staff. The samples tested positive for IgM/IgA and negative for IgG in 5.56%, 13.75%, 0.0%, 0.0%, and 12.50% of the physicians, MLTs, pharmacists, IC staff, and administration staff, respectively. Further, the samples tested positive for IgG and negative for IgM/IgA in 16.67%, 2.50%, 0.0%, 33.33%, and 12.5% of the physicians, MLTs, pharmacists, IC staff, and administration staff, respectively. Finally, the samples tested positive for both IgG and IgM/IgA in 5.56%, 3.75%, 0.0%, 0.0%, and 0.0% of the physicians, MLTs, pharmacists, IC staff, and administration staff, respectively (Table 2). 

No significant (χ2 = 9.42, p = 0.224) group differences were observed in the percentage of the total positive samples among the HCWs from different occupations when the pharmacists were excluded.

## Discussion

To date, the common clinical symptoms and signs, as well as the immune responses, have not been well recognized in individuals with asymptomatic SARS-CoV-2. Our study data showed that 24.24% (80/330) of these HCWs had asymptomatic infections. Thus, asymptomatic COVID-19 infection among HCWs may become a risk factor for patients (other than COVID-19 patient, if HCWs moved from one ward or department to another), family, colleagues, and the community. Therefore, to avoid cross-infection, identification and isolation of asymptomatic carriers among HCWs is important, as well as maintaining a low threshold for suspicion of infection that would control of transmission between HCWs such as close contact with a suspected or confirmed case of COVID-19, or symptoms of SARS-CoV-2 in the past 14 days [12]. Based on the fact that asymptomatic infections were identified in HCWs who were at high risk for infection and not within a random sample of people. Thus, the result of this study might not be an accurate estimation of the proportion of asymptomatic infections in the general population.

In this study, we found that the 6.36% of HCWs included in this study were positive for IgG and negative for IgM/IgA, 14.55% were positive for IgM/IgA and negative for IgG, 3.33% were positive for both IgM/IgA and IgG, while 75.76% were negative for both IgM/IgA and IgG antibodies. According to Jacofsky et al. [13], when the subject is asymptomatic at the time of testing, an IgG seropositive sample indicates that the subject was infected several weeks ago and consequently the immune system had produced antibodies to target the viral antigen. An IgM seropositive sample indicates that the immune system is actively producing antibodies against a recent infection. A sample seropositive for both IgM and IgG indicates that the subject’s immune system is actively producing antibodies against an ongoing infection that likely began more than 14 days ago, whereas a sample negative for both IgM and IgG indicates that the subject is not suspected of having COVID-19, since the immune system has not produced any antibodies to target the viral antigen [14]. However, a subject who is seropositive for IgM, IgG, or both may still be able to spread the disease despite being asymptomatic. Therefore, such individuals should immediately isolate themselves from healthy individuals [12]. To accurately estimate the asymptomatic proportion, serological tests and RT-PCR should be used in conjunction at an appropriate time [15].

In our study, we found that the prevalence of infection was almost equal among all workers in the various departments covered by this study, with the exception of pharmacy staff, suggesting that pharmacy personnel are not at a high risk of infection unless they have contact with a patient’s bodily fluids or are involved in direct patient care [16]. However, the increase in the rate of seropositivity among non-first-line HCWs may be due to a lack of commitment to personal protective equipment (PPE), or may be due to the non-follow-up of infection control teams to these departments, in favor of a focus on frontline HCWs.

The findings of this study have to be seen in light of some limitations. The first limitation concerns the number of COVID-19 patients recorded in Madinah region, as it reached 12,553 on the last day of sample collection for this study. The second limitation is that the increase in the rate of seropositivity among HCWs in this study may be related to the timing of the examination, as the first sample of this study was collected 39 days after the first COVID-19 case was recorded in Madinah region. The third limitation is the sensitivity and specificity of various antibody test kits that may restrict serological tests. Finally, antibodies that were previously found may be a result of infection with human common cold coronaviruses (HCoV-229E, HCoV-NL63, HCoV-OC43, and HCoV-HKU1) or other more severe coronaviruses (SARS-CoV and MERS-CoV).

## Conclusions

In conclusion, the seroprevalence of healthcare workers of five Madinah region-affiliated hospitals was 24.24%. In addition, there were no significant differences observed in the seroprevalence among the different occupations of the HCWs (excluding the pharmacists) with respect to the percentage of their seropositive samples. The outcomes of our study may help in evaluating the efficiency of the current preventive measures for future infection control and occupational health practices. Moreover, the outcomes may also help in predicting the ongoing risk of infection among the vulnerable and enclosed populations.
